# CO_2_ Enrichment Differentially Upregulated Sugar, Proline, and Polyamine Metabolism in Young and Old Leaves of Wheat and Sorghum to Mitigate Indium Oxide Nanoparticles Toxicity

**DOI:** 10.3389/fpls.2022.843771

**Published:** 2022-05-03

**Authors:** Ibrahim I. Shabbaj, Mahmoud M. Y. Madany, Mansour A. Balkhyour, Abdurazag Tammar, Hamada AbdElgawad

**Affiliations:** ^1^Department of Environmental Sciences, Faculty of Meteorology, Environment and Arid Land Agriculture, King Abdulaziz University, Jeddah, Saudi Arabia; ^2^Department of Botany and Microbiology, Faculty of Science, Cairo University, Giza, Egypt; ^3^Department of Biology, College of Science, Taibah University, Medina, Saudi Arabia; ^4^Integrated Molecular Plant Physiology Research, Department of Biology, University of Antwerp, Antwerp, Belgium

**Keywords:** indium oxide nanoparticles, osmoregulation, polyamine metabolism, primary metabolism, sorghum, sugar metabolism, wheat

## Abstract

Soil contamination with indium oxide nanoparticles (In_2_O_3_-NPs) is a challenge for plant growth and productivity. Despite In_2_O_3_-NPs toxicity, their effects on plant growth and metabolism are largely unknown, particularly under future climate CO_2_ (eCO_2_). Therefore, the In_2_O_3_-NPs toxicity and stress mitigating impact of eCO_2_ in the young and old leaves of C3 (wheat) and C4 (sorghum) plants were investigated. Overall, In_2_O_3_-NPs significantly retard the biomass and photosynthetic machinery of all tested crops, particularly the young leaves of C3 plants. Consequently, In_2_O_3_-NPs altered C and N metabolism in C3 and C4 plants. On the other hand, eCO_2_ contrarily alleviated the hazardous effects of In_2_O_3_-NPs on growth and photosynthesis, especially in the young leaves of C4 plants. Increased photosynthesis consequently enhanced the soluble sugars’ accumulation and metabolism (e.g., sucrose P synthase, cytosolic, and vacuolar invertase) in all stressed plants, but to a greater extent in C4 young leaves. High sugar availability also induced TCA organic and fatty acids’ accumulation. This also provided a route for amino acids and polyamines biosynthesis, where a clear increase in proline biosynthetic enzymes [e.g., pyrroline-5-carboxylate synthetase (P5CS), ornithine aminotransferase (OAT), Pyrroline-5-carboxylate reductase (P5CR), pyrroline-5-carboxylate dehydrogenase (P5CDH), and proline dehydrogenase (PRODH)] and polyamine metabolic enzymes (e.g., spermine and spermidine synthases, ornithine decarboxylase, and adenosyl methionine decarboxylase) were mainly recorded in C4 young leaves. The observed increases in these metabolites involved in osmo- and redox-regulation to reduce In_2_O_3_-NPs induced oxidative damage. Overall, our study, for the first time, shed light on how eCO_2_ differentially mitigated In_2_O_3_-NPs stress in old and young leaves of different species groups under the threat of In_2_O_3_-NPs contamination.

## Introduction

Indium, a lustrous silver-white metal, is broadly distributed in the hydrosphere. It shows stability in the air, however, it turns into indium oxide (In_2_O_3_) after exposure to heat. At industrial levels, In_2_O_3_ and In-sulfides are extensively used in electronics production due to their fluorescent and photoconductivity functions (Losev, 2017). Bulk productions of In, however, resulted in their high release in nature through industrial waste (Cummings et al., 2016). Similar to their bulk counterpart, In_2_O_3_ nanoparticles (NPs) represent an environmental challenge for all living organisms. In_2_O_3_-NPs are extensively used in the manufacturing of electronics products (Golan et al., 2007). In_2_O_3_-NPs often contain heavy metals, thus their accumulation has become an important environmental issue. A few studies have investigated In_2_O_3_ NPs toxicity (Jeong et al., 2016; Ahamed et al., 2017), however, their phytotoxic hazards upon plants are little studied.

Nanoparticles attach to plant surface, which imparts plant organ damage. They can also enter the plants through lateral roots junctions (Dietz and Herth, 2011), hydathodes, flower stigmas, and stomata. Moreover, the small sizes of NPs can effectively cross the cell wall and reach the plasma membrane. Consequentially, bioaccumulation of NPs in plants has been reported to influence plant growth (Parthasarathi, 2011), the extent of which depends on the NPs concentration, size, and shape (Stampoulis et al., 2009). This was explained by the decrease in photosynthesis (Shaw et al., 2014) and the increase in NP-mediated DNA damage (Atha et al., 2012). NPs are deposited on the surface of cell and in the organelles that induce oxidative stress signaling (Buzea et al., 2007) including reactive oxygen species (ROS) production (He et al., 2018), disturbance of ion cell membrane transport activity (Auffan et al., 2008), and oxidative damage (lipid peroxidation) (Nouairi et al., 2009). Overall, NPs induced ROS overaccumulation that in turn damaged cell membrane, photosynthetic apparatuses, DNA, and protein biomolecules damages (Atha et al., 2012).

The change in the climate, such as increasing atmospheric CO_2_ levels, will luckily affect plant responses to environmental heavy metal toxicity (Shabbaj et al., 2021a). Atmospheric CO_2_, currently about 400 ppm, may surpass 700 ppm by the end of this century (Long et al., 2004). This increase is mainly due to high CO_2_ emissions from fossil fuels’ combustion, gas flaring, deforestation, and biomass burning. In general, increased CO_2_ (eCO_2_) levels have a fertilizing effect, particularly in C3 plants (AbdElgawad et al., 2015a,b), most likely due to photosynthesis enhancement and water use efficiency (Albert et al., 2011). Moreover, eCO_2_ increased photosynthesis will consequently alter the whole plant metabolism (Selim et al., 2021a). Under control conditions, eCO_2_ mainly affected the physiology and metabolism of the plants, mostly addressing changes in photosynthesis, biomass production, and nutrient relations (De Souza et al., 2008). On the other hand, several studies have reported that eCO_2_ reduced the impact of environmental stresses on plant growth and metabolism (Bauweraerts et al., 2013). For instance, eCO_2_ mitigated the toxicity of several heavy metals and their nanoparticles on plant growth and metabolism (Saleh et al., 2019; Shabbaj et al., 2021a). Under eCO_2_, adding more resources (carbon) will redirect the plant metabolism toward the production of stress-related primary and secondary metabolites. In this regard, enhancing the photosynthetic C assimilation by eCO_2_ improved the non-structural sugars’ accumulation and breakdown via dark respiration (Dusenge et al., 2019). Consequentially, this provides the required precursors and metabolic energy for the synthesis of the different classes of metabolites such as osmo-protectants and antioxidants. In this context, increased sucrose biosynthesis and induced proline catabolism provide high energy to resume stressed plant growth under eCO_2_ (AbdElgawad et al., 2020). Moreover, improved metabolism led to enhanced plant potential in maintaining ROS production and trapping (Pérez-López et al., 2009). Despite the stimulatory effect of eCO_2_ on plant growth and photosynthesis under heavy metals and their nanoparticles, how plant primary and secondary metabolisms are influenced in response to eCO_2_ under In_2_O_3_ NP remains unclear.

Species- and leaf stage- specific are factors influencing plant response to environmental stress, future climate CO_2_, and their interactions. Under control conditions, the growth of C4 species is known to be less affected by the concentration of the current CO_2_ (AbdElgawad et al., 2020). This can be explained by the fact that photosynthesis of C4 plants is almost saturated under ambient CO_2_ levels, which led to reduced photorespiration (Bräutigam and Gowik, 2016). On the other hand, eCO_2_ enhances plant growth and photosynthetic rates under non-stressful conditions, particularly in C3 species (AbdElgawad et al., 2020). However, C3 plants showed more decreases in their photosynthesis and more increases in photorespiration and respiration compared to C4 plants grown under Hg stress (AbdElgawad et al., 2020). Moreover, eCO_2_ differentially mitigated heavy metal-induced stress in C3 and C4. For instance, in wheat (C3) plants, eCO_2_ reduced H_2_O_2_ production, however, in sorghum (C4), eCO_2_ upregulated the ascorbate and glutathione-mediated antioxidative defense system (AbdElgawad et al., 2021). Therefore, it is necessary to understand the eCO_2_ impact on the species-specific responses to soil contamination with In_2_O_3_-NPs (AbdElgawad et al., 2021).

We hypothesize that soil contamination with In_2_O_3_-NPs will negatively impact plant growth. On the other hand, increasing the atmospheric CO_2_ will improve growth, including mitigation of In_2_O_3_-NPs stress impact. Moreover, such effects of eCO_2_ vary considerably between species-groups. Thus, here we aim to first compare the responses of sorghum (C4) and wheat (C3) leaves at different ages to In_2_O_3_-NPs toxicity in terms of growth and physiological and biochemical parameters, second to reveal whether eCO_2_ can effectively mitigate the In_2_O_3_-NPs toxicity, and third whether this mitigation is leaf stage and species group dependent. To this end, we aim to investigate the effect of In_2_O_3_-NPs and/or eCO_2_ on young and old leaves of sorghum and wheat biomass, photosynthesis, primary (e.g., sugar, organic acids, amino acids, and fatty acids) and secondary (e.g., polyamines) metabolism, and oxidative damages. Meanwhile, to validate the potential differential responses, a principal component analysis (PCA) for the all measured data was performed.

## Materials and Methods

### Preparation of Indium Oxide Nanoparticles

In_2_O_3_-NPs were purchased from American elements^1^. In_2_O_3_-NPs are highly pure (99.99%), yellow spherical nano-powder with a specific surface area of 50 m^2^g^–1^ and a bulk density of 7.18 g/cm^3^. In_2_O_3_-NPs size was less than 50 nm. These morphological features were validated (scanning electron microscope, SEM, JEOL JSM-6510). To avoid coarse aggregation of In_2_O_3_ in aqueous solution, NPs were sonicated.

### Plant Materials and Greenhouse Pot Experiment

To better understand the effects of In_2_O_3_-NPs on plants, this study was performed with conditions that approach environmentally realistic conditions. The seed of *Sorghum bicolor* (L. cv. Giza 3) (C4) and *Triticum aestivum* L., cv. Giza 155 (C3) were obtained. They are a commercial high yielding hybrid variety grown in large areas of Egypt, moreover, this variety showed moderate tolerance to heavy metal stress, so they have been used as a model. Healthy seeds were sterilized in a solution of Na-hypochlorite (5% v/v, 25 min) and washed thoroughly with distilled water. The sterilized seeds were grown in 15 cm diameter, 30 cm height polyvinyl chloride (PVC) tubes containing sandy soil (90%) sand at an adjusted pH 7.6. The soil initially contained 1.46% carbon, 23.6 mg nitrate-nitrogen (N), 1.25 mg ammonium-N, and 15.8 mg phosphorus kg^–1^ air. The soil was daily watered to adjust the water soil capacity at 68%. Plants were grown in sunlit CO_2_ and temperature-CO_2_-controlled chambers. The top of sunlit chambers consisted of a transparent polycarbonate plate to allow light. The temperature was adjusted at 26/20°C (day and night) and the Photosynthetic Active Radiation (PAR) was measured by a SDEC, type JYP1000 quantum sensor. The healthy seeds of C3 (wheat) and C4 (*Sorghum*) were grown under four growth conditions: (1) current CO_2_ (aCO_2_, 385 ppm); (2) aCO_2_ + In_2_O_3_ (250 mg/kg soil); (3) future climate CO_2_ (eCO_2_, 665 ppm); and 4) eCO_2_ + In_2_O_3_ (250 mg/Kg soil). After 7 weeks of growth, the rhizosphere soil as well as plant samples, i.e., [1, 2, 3, and 4th leaf (old tissues) and 5, 6, and 7th leaf (young tissue)] were collected and kept in −80°C for further analysis. The fresh and dry weight of roots and shoots were determined.

### Measurement of Photosynthetic Activity

Photosynthetic rate (μmol CO_2_ m^–2^ s^–1^) (Asat, the light-saturated leaf-level) was measured by LI-COR LI- 6400, LI-COR Inc., following the method described by Miner et al. (2017). The photosynthetic-related enzyme [e.g., ribulose-1,5-bisphosphate carboxylase/oxygenase (RuBisCo)] was measured according to Sulpice et al. (2007).

### Sugar Metabolism

Sugars were extracted in 50 mM TAE buffer pH 7.5 containing 0.02% Na-azide, 10 mM mannitol, 0.15% polyclar, 12 mM NaHSO_3_, 1 mM mercapto-ethanol, and 2 mM PMSF. The extracts were centrifuged at 15,000 *g* and 4°C, for 10 min. Part of the extract was heated for 5 min at 90°C and, after cooling and centrifugation at 14,000 *g* (4°C and 5 min), the supernatants were transferred to mixed bed Dowex column of 300 μl Dowex H^+^, 300 μl Dowex Ac^–^; both 100–200 mesh. After elution with ddH_2_O, glucose, fructose, sucrose, and raffinose concentrations were measured (HPAEC-PAD) (Vergauwen et al., 2000). Non-heated supernatant was used for measuring the activity of the key sugar enzymes (Madany and Khalil, 2017). The activity of the invertase enzymes was measured In TAE buffer with 100 mM sucrose. Reaction mixtures were incubated at 30°C, and the reactions were stopped using an aliquot for 5 min in a water bath at 90°C. Glucose and fructose concentrations were measured as described above.

The activity of sucrose phosphate synthase (SPS) was measured in 1 ml of HEPES buffer at pH 8 contained UDP- glucose and mM fructose-6-phosphate at 37°C for 20 min and stopped by adding NaOH (30%). The reaction mixture containing citrate and glycogen initiated the starch synthase (Nishi et al., 2001). A 0.2%v starch solution containing 0.05% of I2/KI (w/v) in 0.05% HCl was used to detect amylase activity at 620 nm (Madany and Khalil, 2017).

### Amino Acids Metabolism

Amino acids were extracted in 2 ml of 80% (v/v) aqueous ethanol, spiked with norvaline as internal standard, and the homogenate was centrifuged at 14,500 rpm for 18 min. The supernatant was vacuum evaporated, and the pellet resuspended in 1 ml chloroform. Simultaneously, residue was re-extracted with 1 ml HPLC-grade water and, after centrifugation at 14,000 rpm for 20 min, the supernatant was mixed with the pellet suspended in chloroform. The combined extract was centrifuged for 10 min at 14,000 rpm and the aqueous phase was filtered through Millipore micro filters (0.2 μM pore size) before assaying amino acids levels. All amino acid individuals were determined by using a Waters Acquity UPLC-tqd system equipped with a BEH amide 2.1 × 50 column (Sinha et al., 2013).

The activities of key enzymes involved in proline metabolism, i.e., pyrroline-5- carboxylate synthetase (P5CS), pyrroline-5-carboxylate reductase (P5CR), pyrroline-5-carboxylate dehydrogenase (P5CDH), proline dehydrogenase (ProDH), ornithine amino transferase (OAT), and arginase (Arg), were extracted [Tris-HCl buffer (50 mM, pH 7.4)] (Shabbaj et al., 2021b). The activities of OAT and ARG were assessed in KPO4 buffer (50 mM KPO4 buffer pH 7.0). The activities of all enzymes (P5CS, P5CDH, PRODH, OAT, GS, and ARG) were measured by monitoring the reduction of NADH at A340 (Shabbaj et al., 2021b), the P5C-dependent NADH oxidation at A340 (Lutts et al., 1999), production γ-glutamyl hydroxamate at A535 (Temple et al., 1996), the reduction of 2,6-dichloroindophenol at A600 (Chen et al., 2004), the production by using the method of diacetyl monoxime described by Chen et al. (2004), and the glutamine-dependent NADH oxidation at A340 (Robinson et al., 1991). By using BSA as a standard, the protein concentrations were measured as assessed by Lowry et al. (1951).

### Fatty Acids Metabolism

Fatty acids were quantified using GC mass (Madany et al., 2020), where a weight of 0.5 g of LN-fine powdered plant leaves were extracted in aqueous methanol at 27°C. The GC/MS analysis was performed (Hewlett Packard 6890, MSD 5975 mass spectrometer, Hewlett Packard, Palo Alto, CA, United States) with HP-5 MS column of 30 m × 0.25 mm × 0.25 mm. The fatty acids were calculated from the standard curve using the analyte/internal standard ion yield ratios.

### Polyamine Metabolism

Plant samples were extracted in cold perchloric acid (Bregoli et al., 2002). After centrifugation for 30 min at 14,000 *g*, we applied dansyl chloride to derivatize the samples supernatants and standards in order to detect free polyamines. After hydrolyzation at 110°C overnight, in 6 N HCl was added for the formation of conjugated polyamines. The reverse phase HPLC (Shimadzu SIL10-ADvp; C18 column) was used to measure the concentrations of dansylated polyamine derivatives. For measuring the activities of polyamine related enzymes, plant shoot and roots were homogenized in 100 mM KPO4, pH 7.5, and they were centrifuged for 20 min at 25,000 RPM. Ornithine decarboxylase enzyme (ODC) and arginine decarboxylase (ADC) enzyme activity were measured by measuring the labeled CO_2_ (Birecka et al., 1985), which was liberated from L-[l-14C] Ar (55 mCi mmol^–1^) and L-[l-14C] Orn (55 mCi mmol^–1^). The trapped radioactivity was measured in a liquid scintillation counter. The activity of spermidine synthase (SpdS) was assayed in a reaction mixture of pH 8.0 Tris-HCl (0.1 M) (Yoon et al., 2000). The production of 5′-deoxy-5′-methylthioadenosyne was quantified by using a fluorescence detection method (a reverse phase HPLC equipped with a fluorescence detector). Spermine synthase (SpmS) activity was measured by the production of methylthioadenosine (Cason et al., 2003).

### Statistical Analysis

All results were expressed as the mean of five biological replicates (*n* = 5). Statistical analysis was performed using one-way ANOVA in the SPSS 22 (Tukey test, *P* ≤ 0.05). Data normality was checked by using Levene’s test. Hierarchical cluster analysis Euclidean distance was performed using the R stat software package (version 4.5.0, the R).

## Results

### Elevated CO_2_ Highly Improves the Biomass and Photosynthetic Efficiency of Sorghum and Wheat Grown Under Indium Oxide Nanoparticles Conditions

**FIGURE 1:**
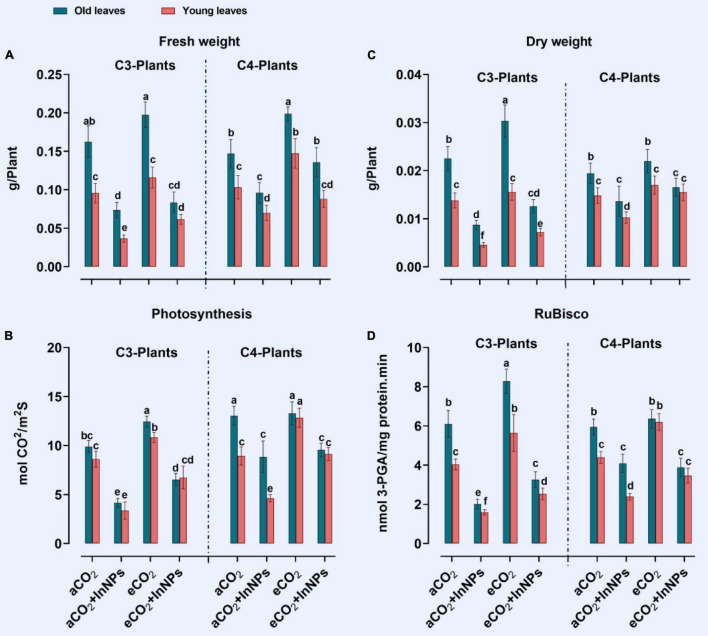
**(A–D)** Effect of the co-application of future climate CO_2_ and In_2_O_3_-NPs upon the biomass (FW and DW) and photosynthesis of both old and young leaves of C3 and C4 plants. Four biological replicates are used to measure each value. The bars on the columns represent error bars (SE). One way ANOVA test (P < 0.05; n = 4) was applied to show the significant difference between groups. Different letters showed the significant difference between treatments.

Previously, it was proved that indium adversely affects the growth and development of higher plants (Syu et al., 2017). The treatment of C3 plants with In_2_O_3_-NPs reduced biomass accumulation (FW, DW), photosynthesis, and RuBisco activity in young leaves by about 30, 14, 50, and 65%, respectively, in comparison to control plants (Figures 1A–D). Like C3 plants, old leaves of C3 plants treated with In_2_O_3_-NPs reduced biomass accumulation (FW and DW), RuBisco activity (by about 20%), and photosynthesis (by 40%) as compared with control plants. Additionally, the young leaves of C3 plants suffered from a remarkable reduction in their FW, DW, photosynthesis, and RuBisco (60, 50, 60, and 80% reduction) in response to treatment with In_2_O_3_-NPs. On the other hand, extreme levels of CO_2_ were reported to alleviate the adverse effects of different environmental conditions (Shabbaj et al., 2021a). In this context, the exposure of C4 plants to eCO_2_ significantly augmented the biomass (FW and DW) as well as the photosynthetic related parameters (photosynthesis and RuBisco). A further enhancement in the biomass was observed in the old leaves of C4 plants in which the increment reached 40 and 100% in FW and DW, respectively (Figure 1). Similarly, a noticeable improvement in the FW and DW as well as the photosynthesis and RuBisco activity was observed upon treatment with eCO_2_ either alone or in combination with In_2_O_3_-NPs as compared with their counter control (Figure 1). Moreover, elevated levels of CO_2_ caused a slight increase in the biomass and photosynthesis of young leaves C3 plants (∼10–20% increase) as compared to their respective controls. Consistently, CO_2_ enrichment was found to augment the FW, DW, photosynthesis, and RuBisco activity of C4 plants (at the young leaves), that reached up to 40% when compared to their counter control plants.

### Effect of Future Climate Upon Osmoregulatory Status of Both Sorghum and Wheat Grown in Indium Oxide Nanoparticles Contaminated Soils

#### Sugar Metabolism

**FIGURE 2:**
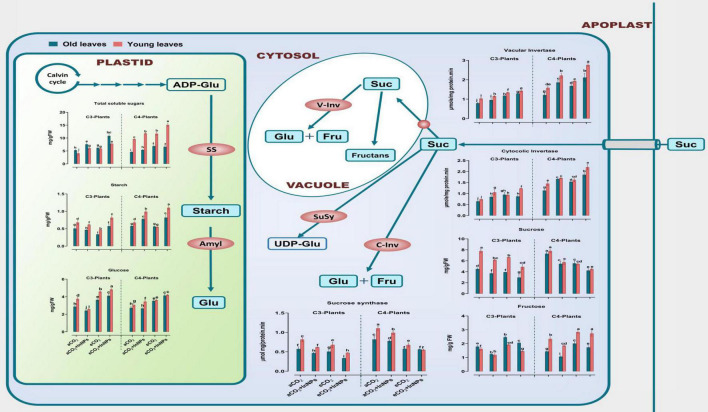
Effect of the co-application of future climate CO_2_ and In_2_O_3_-NPs upon the sugar metabolism in both old and young leaves of C3 and C4 plants. Four biological replicates are used to measure each value. The bars on the columns represent error bars (SE). One way ANOVA test (*P* < 0.05; *n* = 4) was applied to show the significant difference between groups. Different letters showed the significant difference between treatments.

Heavy metals greatly affect sugar metabolism in higher plants (Selim et al., 2021b). The current results showed that both old and young leaves of C3 and C4 plants respond differently to the effects of eCO_2_ and/or In_2_O_3_-NPs on the levels of sugars (i.e., starch, glucose, sucrose, fructose, and total sugars; TS) and the sugar-related enzymes (cytosolic invertase; C-Inv, vacuolar invertase; V-Inv as well as sucrose synthase; SuSy) (Figure 2). Old and young leaves of C3 plants seem to be equally affected by the individual treatment with In_2_O_3_-NPs, causing significant elevations in the levels of sucrose and TS (∼30–40% increase), as well as the activity of cyto-inv as compared with the untreated control plants. On the other hand, both starches, glucose and fructose, were significantly decreased when compared with their counter control plants. However, the activity of SuSy was much more enhanced (by about 100%) in old leaves in response to treatment with In_2_O_3_-NPs relative to the untreated control plants (Figure 2). On the other side, both old and young leaves of C3 and C4 plants respond differently to the application of eCO_2_ either individually or in combination with In_2_O_3_-NPs. For instance, the activity of C-Inv exhibited a noticeable enhancement in response to eCO_2_ either solely or in combination with In_2_O_3_-NPs, particularly in the old and young leaves of C4 plants as compared with their respective control plants (Figure 2). On the other hand, the application of eCO_2_ and/or In_2_O_2_-NPs caused a reduction in the levels of sucrose and starch in both old and young leaves of C3 and C4 plants relative to their counter control plants. Moreover, it was also reported that glucose, fructose, and TS were increased at both old and young leaves (by about 20–50%) in response to eCO_2_ either alone or in combination with In_2_O_3_-NPs when compared with their respective control plants.

On the other hand, old and young leaves of C4 plants have shown a partially similar response to the sole treatment with In_2_O_3_-NPs, whereas remarkable increases were observed in sucrose, TS, SuSy, and cyto-inv, while glucose and fructose were significantly reduced, when compared to the non-treated control. Likewise, the individual treatment of C4 plants (at both old and young leaves) with eCO_2_ positively affected the levels of fructose, sucrose, TS, and cyto-inv (increased by 20–50%), while no significant changes were observed for SuSy and glucose in old and young leaves, respectively. Furthermore, both stages have responded differently to the combined treatment with In_2_O_3_-NPs and eCO_2_, leading to elevations in all the detected sugars and their-related enzymes, whereas the level of SuSy was much more enhanced at the young leaves (increased by 110%), when compared with control plants.

#### Organic Acids’ Metabolism

**FIGURE 3:**
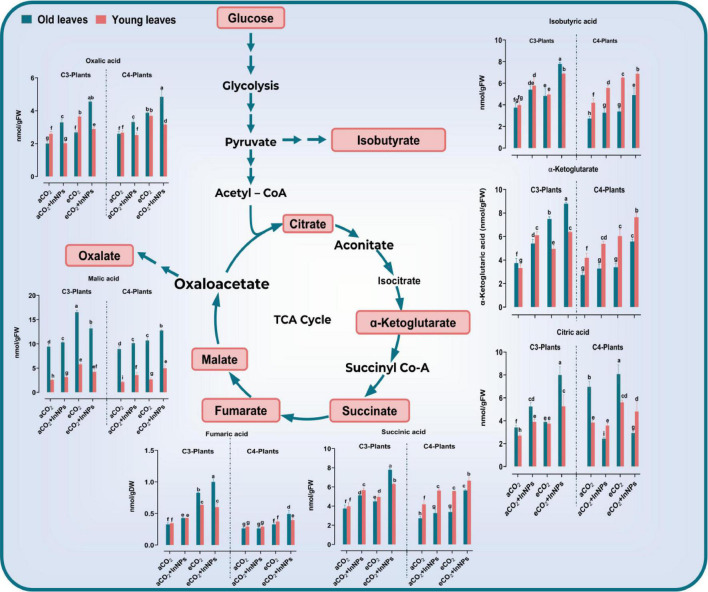
Effect of the co-application of future climate CO_2_ and In_2_O_3_-NPs upon the accumulation of organic acid in both old and young leaves of C3 and C4 plants. Four biological replicates are used to measure each value. The bars on the columns represent error bars (SE). Fisher’s LSD test (*P* < 0.05; *n* = 4) was applied to show the significant difference between groups. Different letters showed the significant difference between treatments.

It was reported that organic acids contributed mainly to enhancing the plant tolerance against environmental stresses, particularly heavy metals (Osmolovskaya et al., 2018). In the present study, the levels of organic acids (oxalic, succinic, fumaric, malic, isobutyric, and citric acids) were analyzed in old and young leaves of C3 and C4 plants to show the effect of In_2_O_3_-NPs and/or eCO_2_ (Figure 3). Here, we found that both C3 and C4 respond differently to the individual treatment with In_2_O_3_-NPs. For example, In_2_O_3_-NPs had no remarkable effect upon the levels of oxalate in young leaves of C3 and C4 species as well as fumarate, citrate, and α-ketoglutarate in old and young leaves in C4 plants. However, In_2_O_3_-NPs caused a significant increment in the levels of other measured organic acids in both old and young leaves of C3 and C4 crops (Figure 3). Such an enhancing effect was more pronounced in the levels of oxalate, isobutyrate, and citrate in C3 plants that reached to about 60%, when compared with their respective control plants (Figure 3). On the other hand, the individual treatment with eCO_2_ caused further increment in the levels of organic acids in both old and leaves of C3 and C4, particularly succinate and fumarate (by about 80–110% increase), as well as malate, isobutyrate, and citrate (by about 20–50% increase) as compared with their respective control plants. Similarly, both old and young leaves of C3 and C4 species responded positively and equally to the combined treatment with In_2_O_3_-NPs and eCO_2_ by increasing the levels of malate (by about 30%), isobutyrate, and citrate (by 80–110%), as well as α-ketoglutarate, succinate, and fumarate which exhibited a noticeable increment especially in young leaves of C4 plants as compared with control plants (Figure 3).

#### Amino Acids’ Profile

As organic acids represent the stored pool of fixed carbons, their levels will accordingly affect the levels of amino acids’ pool in higher plants (Igamberdiev and Eprintsev, 2016). Therefore, we concomitantly investigated the effect of eCO_2_ and/or In_2_O_3_-NPs upon the levels of several amino acids in old and young leaves of C3 and C4 plants (Table 1). Overall, the majority of the measured amino acids exhibited a remarkable accumulation in response to individual treatment with In_2_O_3_-NPs, particularly glycine, phenylalanine, valine, and serine, which were much more increased in the young leaves than the old ones (∼40–120% increase), as well as alanine that was more enhanced in the old ones (∼90% increase), when compared with their counter control plants (Table 1). On the other hand, a noticeable reduction has been observed in ornithine, asparagine, isoleucine, leucine, methionine, glutamic acid, cystine, and tyrosine in the old leaves of C3 species as compared with untreated control plants (Table 1). Therefore, it could be noted that the old leaves of C3 plants were more sensitive to In_2_O_3_-NPs than the young leaves. Meanwhile, both old and young leaves have positively interacted to the individual effect of eCO_2_ on all the detected amino acids, sharing similar responses regarding the increments, particularly in glycine, histidine, ornithine, arginine, and cysteine in comparison to their respective control plants. Additionally, the co-application of eCO_2_ and In_2_O_3_-NPs caused further accumulation in the majority of the measured amino acids in both old and young leaves of C3 plants when compared with untreated and treated control plants (Table 1). C3 On the other hand, the levels of amino acids responded differently to the individual treatment of In_2_O_3_-NPs in both old and young leaves of C4 plants (Table 2). An observable increase was detected in the levels of arginine, ornithine, and aspartate (by about 20–50%), leucine (by 200%), and serine and phenylalanine (by about 80–100%) in the young leaves of C4 plants as compared with the untreated control plants. Meanwhile, a remarkable decline was observed in the levels of glutamine, asparagine, isoleucine, methionine, threonine, and valine in both old and young leaves of C4 plants (Table 2). A further increment was detected in almost all amino acids in response to the individual treatment with the eCO_2_. Moreover, the co-application of In_2_O_3_-NPs and eCO_2_ positively influenced both old and young leaves of C4 plants by augmenting the contents of most detected amino acids, except for lysine, valine, and cysteine that were significantly reduced in the old leaves (Table 2). Thus, it is clear that the eCO_2_-induced effect was more pronounced in old leaves, while young leaves were more responsive to the combined effect of both In_2_O_3_-NPs and eCO_2_ than the old leaves. Overall, C4 plants were more responsive than C3 plants to the combined effect of eCO_2_ and In_2_O_3_-NPs, thus enhancing their amino acids contents.

**TABLE 1:** The effect of the co-application of future climate CO_2_ and In_2_O_3_-NPs upon the amino acids profile of both old and young leaves of C3 plants.

	C3 Plants							
	Old leaves				Young leaves			
	aCO_2_	aCO_2_ + In_2_O_3_-NPs	eCO_2_	eCO_2_ + In_2_O_3_-NPs	aCO_2_	aCO_2_ + In_2_O_3_-NPs	eCO_2_	eCO_2_ + In_2_O_3_-NPs
Glycine	50.42 ± 2.21^a^	63.07 ± 2.63^a^	77.72 ± 4.25^b^	78.58 ± 9.53^b^	59.17 ± 2.64^a^	74.97 ± 3.61^b^	79.77 ± 3.86^b^	84.45 ± 0.14^c^
Lysine	3.45 ± 0.45^a^	4.12 ± 0.12^a^	4.26 ± 0.21^a^	4.78 ± 0.11^ab^	3.81 ± 0.35^a^	3.92 ± 0.33^a^	4.04 ± 0.32^a^	4.21 ± 0.31^a^
Histidine	1.76 ± 0.36^b^	0.68 ± 0.12^a^	1.92 ± 0.04^b^	1.65 ± 0.42^b^	1.72 ± 0.51^b^	1.42 ± 0.26^b^	1.91 ± 0.04^b^	1.91 ± 0.61^b^
Alanine	8.28 ± 0.20^a^	14.86 ± 0.84^b^	11.37 ± 0.31^ab^	15.66 ± 0.81^b^	9.77 ± 0.31^a^	13.99 ± 0.61^b^	16.31 ± 0.71^b^	14.86 ± 0.51^b^
Arginine	1.06 ± 0.10^a^	0.85 ± 0.82^a^	1.53 ± 0.51^b^	1.15 ± 0.08^a^	1.13 ± 0.46^a^	0.85 ± 0.02^a^	1.26 ± 0.21^b^	1.46 ± 0.41^b^
Ornithine	0.89 ± 0.58^c^	0.45 ± 0.43^ab^	1.11 ± 0.05^d^	0.67 ± 0.31^b^	0.31 ± 0.11^a^	0.36 ± 0.02^a^	0.35 ± 0.01^a^	0.38 ± 0.08^a^
Glutamine	2.29 ± 1.27^b^	1.53 ± 0.51^a^	3.63 ± 0.61^c^	1.58 ± 0.11^a^	1.82 ± 0.42^ab^	2.27 ± 0.31^b^	2.23 ± 0.12^b^	2.52 ± 0.31^b^
Asparagine	3.41 ± 0.41^c^	1.41 ± 0.05^b^	3.81 ± 0.41^c^	1.58 ± 0.41^b^	1.14 ± 0.04^a^	1.25 ± 0.61^a^	1.19 ± 0.04^a^	2.13 ± 0.3b^c^
Isoleucine	1.34 ± 0.07^b^	0.15 ± 0.04^a^	1.98 ± 0.03a	0.92 ± 0.01^b^	0.17 ± 0.01^a^	0.21 ± 0.05^a^	0.31 ± 0.01^b^	0.22 ± 0.01^a^
Leucine	0.39 ± 0.01^d^	0.13 ± 0.03^b^	0.75 ± 0.11^e^	0.29 ± 0.07^c^	0.03 ± 0.01^a^	0.18 ± 0.01^b^	0.11 ± 0.03^b^	0.28 ± 0.01^c^
Methionine	0.36 ± 0.12^c^	0.14 ± 0.06^b^	0.58 ± 0.31^d^	0.21 ± 0.02^b^	0.07 ± 0.05^b^	0.03 ± 0.01^a^	0.12 ± 0.05^b^	0.09 ± 0.04^b^
Threonine	0.58 ± 0.02^ab^	0.46 ± 0.03^a^	0.82 ± 0.01^c^	0.78 ± 0.11^c^	0.41 ± 0.21^a^	0.74 ± 0.42^c^	0.62 ± 0.42^b^	0.98 ± 0.21^d^
Valine	0.71 ± 0.05^a^	0.88 ± 0.15^a^	0.93 ± 0.21^ab^	1.21 ± 0.23^b^	0.71 ± 0.06^a^	1.11 ± 0.12^b^	1.25 ± 0.12^b^	1.41 ± 0.06^b^
Serine	0.35 ± 0.01^a^	0.83 ± 0.04^b^	1.07 ± 0.04^b^	1.36 ± 0.11^b^	0.39 ± 0.01^a^	0.89 ± 0.08^b^	0.69 ± 0.01^b^	1.19 ± 0.21^c^
Phenylalanine	0.89 ± 0.18^a^	1.13 ± 0.11^b^	1.35 ± 0.31^b^	1.42 ± 0.31^b^	0.62 ± 0.16^a^	1.51 ± 0.3b^c^	1.07 ± 0.31^a^	1.91 ± 0.51^b^
Glutamic acid	1.14 ± 0.04^b^	0.5 ± 0.042^a^	2.55 ± 0.51^c^	1.01 ± 0.03^b^	0.43 ± 0.02^a^	0.99 ± 0.05^b^	0.96 ± 0.04^b^	2.11 ± 0.54^c^
Aspartate	0.21 ± 0.04^b^	0.14 ± 0.01^a^	0.21 ± 0.02^b^	0.16 ± 0.03^a^	0.25 ± 0.11^b^	0.21 ± 0.01^b^	0.33 ± 0.02^c^	0.45 ± 0.02^d^
Cystine	0.49 ± 0.07^b^	0.15 ± 0.03^a^	0.65 ± 0.21^c^	0.42 ± 0.31^b^	0.62 ± 0.04^c^	0.31 ± 0.12^a^	0.83 ± 0.04^c^	0.39 ± 0.31^b^
Tyrosine	0.81 ± 0.05^c^	0.29 ± 0.02^a^	1.32 ± 0.02^b^	0.73 ± 0.05^c^	0.57 ± 0.04^b^	0.61 ± 0.04^b^	1.34 ± 0.71^b^	1.12 ± 0.06^b^

*Values are represented as the mean of 3 replicates (mean ± S.D.). The different letters indicate significant difference between groups in each row (p < 0.05).*

**TABLE 2:** Effect of the co-application of future climate CO_2_ and In_2_O_3_-NPs upon the amino acids profile of both old and young leaves of C4 plants.

	C4 Plants							
	Old leaves		Young leaves		Old leaves		Young leaves	
	aCO_2_	aCO_2_ + In_2_O_3_-NPs	aCO_2_	aCO_2_ + In_2_O_3_-NPs	eCO_2_	eCO_2_ + In_2_O_3_-NPs	eCO_2_	eCO_2_ + In_2_O_3_-NPs
**Glycine**	64.37 ± 2.41^a^	69.86 ± 2.52^a^	60.62 ± 2.71^a^	68.91 ± 3.31^a^	84.75 ± 4.02^b^	86.71 ± 2.52^c^	61.64 ± 2.51^a^	70.46 ± 0.32^b^
Lysine	10.51 ± 0.32^c^	6.61 ± 0.32^b^	3.74 ± 0.22^a^	3.79 ± 0.61^a^	15.09 ± 0.91^d^	7.51 ± 1.81^b^	4.81 ± 0.61^a^	4.95 ± 0.61^a^
Histidine	1.94 ± 0.31^b^	1.85 ± 0.31^a^	1.35 ± 0.02^a^	2.27 ± 0.41^b^	2.96 ± 0.41^b^	2.71 ± 0.61^b^	2.49 ± 0.41^b^	2.61 ± 0.21^b^
Alanine	12.81 ± 0.50^b^	8.93 ± 0.31^a^	16.72 ± 0.71^b^	23.65 ± 1.11^c^	15.14 ± 0.6^b^	15.35 ± 0.51^b^	19.61 ± 0.81^c^	31.47 ± 1.91^d^
Arginine	0.88 ± 0.50^a^	1.12 ± 0.03^a^	0.84 ± 0.42^a^	1.37 ± 0.11^b^	1.51 ± 0.09^b^	1.49 ± 0.52^b^	1.66 ± 0.21^b^	1.66 ± 0.21^b^
Ornithine	0.45 ± 0.06^a^	0.64 ± 0.39^a^	0.75 ± 0.52^ab^	0.56 ± 0.02^a^	1.33 ± 0.61^c^	0.82 ± 0.12^ab^	0.97 ± 0.18^b^	0.95 ± 0.11^b^
Glutamine	1.49 ± 0.20^b^	1.42 ± 0.11^b^	2.76 ± 0.41^c^	0.67 ± 0.24^a^	2.22 ± 1.11^c^	2.33 ± 1.11^c^	3.56 ± 0.31^d^	1.96 ± 1.16^b^
Asparagine	0.67 ± 0.05^a^	0.66 ± 0.31^a^	1.83 ± 0.42^c^	0.67 ± 0.06^a^	1.12 ± 0.07^b^	0.97 ± 0.09^b^	1.96 ± 0.07^c^	1.36 ± 0.11^b^
Isoleucine	0.19 ± 0.01^a^	0.17 ± 0.06^a^	1.18 ± 0.04^c^	0.26 ± 0.05^a^	1.57 ± 0.05^c^	0.23 ± 0.071^ab^	1.87 ± 0.51^b^	0.35 ± 0.06^b^
Leucine	0.07 ± 0.02^a^	0.21 ± 0.05^b^	0.17 ± 0.04^b^	0.18 ± 0.05^b^	0.28 ± 0.07^b^	0.25 ± 0.06^b^	0.24 ± 0.06^b^	0.23 ± 0.06^b^
Methionine	0.06 ± 0.02^b^	0.02 ± 0.01^a^	0.22 ± 0.08^c^	0.15 ± 0.03^c^	0.36 ± 0.01^d^	0.12 ± 0.031^c^	0.34 ± 0.06^d^	0.32 ± 0.01^d^
Threonine	1.18 ± 0.10^b^	0.83 ± 0.51^a^	0.81 ± 0.51^a^	0.54 ± 0.42^a^	1.58 ± 0.31^b^	0.98 ± 0.51^a^	1.35 ± 0.31^b^	1.21 ± 0.71^b^
Valine	2.52 ± 0.09^b^	1.27 ± 0.04^a^	1.37 ± 0.05^a^	1.09 ± 0.07^a^	2.76 ± 0.11^b^	1.38 ± 0.05^a^	1.41 ± 0.11^a^	1.84 ± 0.06^b^
Serine	0.31 ± 0.08^a^	0.62 ± 0.41^a^	0.34 ± 0.11^a^	0.53 ± 0.22^a^	0.64 ± 0.31^a^	0.86 ± 0.11^b^	0.84 ± 0.02^b^	0.87 ± 0.11^b^
Phenylalanine	1.05 ± 0.84^a^	1.87 ± 0.75^a^	0.91 ± 0.54^a^	1.69 ± 1.05^a^	1.42 ± 0.67^a^	2.06 ± 0.07^b^	1.56 ± 0.44^a^	2.21 ± 0.06^b^
Glutamic acid	1.93 ± 0.06^ab^	1.88 ± 0.01^ab^	1.27 ± 0.04^a^	2.49 ± 0.08^b^	2.63 ± 0.06^a^	2.64 ± 0.51^b^	1.89 ± 0.51^ab^	3.23 ± 0.04^c^
Aspartate	0.21 ± 0.01^a^	0.31 ± 0.01^a^	0.18 ± 0.02^a^	0.36 ± 0.05^b^	0.26 ± 0.03^a^	0.54 ± 0.11^b^	0.27 ± 0.14^a^	0.62 ± 0.21^b^
Cystine	0.65 ± 0.02^b^	0.33 ± 0.21^a^	0.31 ± 0.21^a^	0.42 ± 0.06^ab^	1.16 ± 0.34^a^	0.51 ± 0.41^b^	0.57 ± 0.01^b^	0.63 ± 0.01^b^
Tyrosine	1.38 ± 0.09^a^	0.63 ± 0.04^a^	0.51 ± 0.03^a^	0.59 ± 0.04^a^	2.28 ± 0.1c^a^	1.29 ± 0.02^b^	1.21 ± 0.51^b^	1.25 ± 0.15^b^

*Values are represented as the mean of 3 replicates (mean ± S.D.). The different letters indicate significant difference between groups in each row (p < 0.05).*

#### Proline Metabolism

**FIGURE 4:**
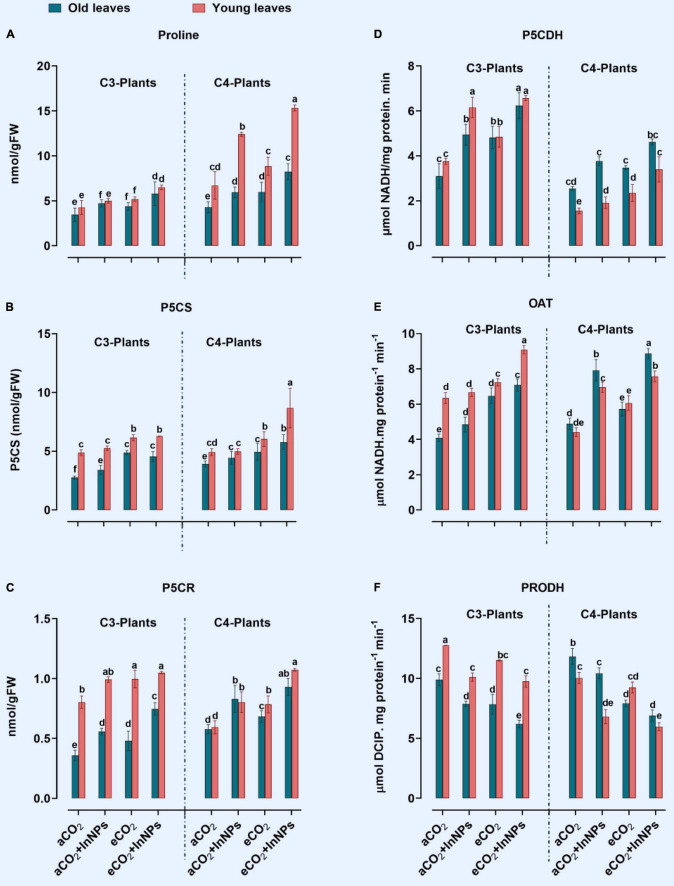
**(A–F)** Effect of the co-application of future climate CO_2_ and In_2_O_3_-NPs upon proline metabolism in both old and young leaves of C3 and C4 plants. Four biological replicates are used to measure each value. The bars on the columns represent error bars (SE). One way ANOVA test (P < 0.05; n = 4) was applied to show the significant difference between groups. Different letters showed the significant difference between treatments.

Similar to other amino acids, proline plays a pivotal role in stabilizing cellular structures and maintaining enzymatic activities (Madany et al., 2020). In the current study, the proline content and proline-related enzymes, which are pyrroline-5-carboxylate synthetase (P5CS), ornithine aminotransferase (OAT), Pyrroline-5-carboxylate reductase (P5CR), pyrroline-5-carboxylate dehydrogenase (P5CDH), and proline dehydrogenase (PRODH), have been investigated in both C3 and C4 plants grown under higher levels of eCO_2_ and/or In_2_O_3_-NPs (Figure 4). In old leaves of C3 plants, there were significant elevations in the levels of proline which positively correlated with the increase in the activity of its biosynthetic enzymes (P5CS, P5CR, and P5CDH), with a concomitant reduction in the activities of its degrading enzymes (PRODH), in response to the individual treatment with In_2_O_3_-NPs (Figures 4A–D). Both old and young leaves of C3 seemed to be equally affected by such treatment, however, the activity of OAT was enhanced only in old leaves, but not in the young leave of C3 plants (Figure 4E). Similarly, both old and young leaves of C3 plants interacted with the combined effects of In_2_O_3_-NPs and eCO_2_.

Similarly, the proline metabolism was affected in both old and young leaves of C4 plants in response to the individual treatment with In_2_O_3_-NPs. In this context, In_2_O_3_-NPs caused a significant elevation in the activities of P5CR and P5CDH (Figures 4C,D). It was also observed that OAT and PRODH were markedly increased in old leaves, while proline content was much more increased (by about 90%) in young leaves of C4 plants (Figures 4E,F). Moreover, eCO_2_ caused a further accumulation in the levels of proline, as well as the activities of OAT, P5CDH, PRODH, P5CS, and P5CR in the old leaves of C4 plants. On the other hand, P5CS was not affected in the young leaves under the individual treatment with In_2_O_3_-NPs. In old and young leaves of C4 plants, the effect of eCO_2_ alone or combination with In_2_O_3_-NPs has increased their contents of proline (∼80–100%) in comparison to control plants (Figure 4A). By comparing C3 and C4 plants, C4 plants have responded better to the combined effect of In_2_O_3_-NPs and eCO_2_ on enhancing their proline content and its related enzymes. Also, the old leaves of C4 plants had higher response to eCO_2_ than those of C3 plants.

#### Fatty Acids Metabolism

Fatty acids are essential not only for membrane integrity but also for plant growth and development (Yaeno et al., 2004). Our results revealed a remarkable variation in the fatty acids’ profile of both C3 and C4 plants under the different effects of In_2_O_3_-NPs and/or eCO_2_ (Table 3). Both old and young leaves of C3 plants have shown a partially similar response to the individual treatment with In_2_O_3_-NPs by reducing the levels of most detected fatty acids such as linoleic, linolenic, myristic, pentacosanoic, eicosenoic, and tricosanoic (reduced by 60–110%) as compared to non-treated controls (Table 3). However, there were significant increases in heptadecenoic (by about 100%) and oleic (by 10%) in the old leaves only. Meanwhile, the individual treatment with eCO_2_ caused a pronounced elevation in the majority of the detected fatty acids; myristic, pentacosanoic, linolenic, and linoleic (increased by 20–50%) and oleic and tetracosenoic acids (60-100%) in both old and young leaves of C3 plants (Table 3). The levels of stearic and palmitoleic acids were markedly reduced in only the young leaves of C3 plants. Additionally, in both old and young leaves, the higher levels of CO_2_ have cooperated with In_2_O_3_-NPs to exert higher increases in the contents of docosanoic, oleic, linoleic (elevated by 20–40%), and tetracosenoic (by about 100%), while heptadecanoic, tricosanoic, pentacosanoic, and eicosenoic were not affected when compared to the non-treated controls (Table 3). The levels of stearic and palmitoleic acids were increased by about 100% in young leaves, while heptadecenoic acid was enhanced only in old leaves. Thus, both old and young leaves could be expected to be equally responsive to a combined impact on their fatty acids’ profiles.

**TABLE 3:** Effect of the co-application of future climate CO_2_ and In_2_O_3_-NPs upon the fatty acids profile of both old and young leaves of C3 plants.

	C3 Plants				C3 Plants			
	Old leaves		Young leaves		Old leaves		Young leaves	
	aCO_2_	aCO_2_ + In_2_O_3_-NPs	aCO_2_	aCO_2_ + In_2_O_3_-NPs	eCO_2_	eCO_2_ + In_2_O_3_-NPs	eCO_2_	eCO_2_ + In_2_O_3_-NPs
Myristic (C14:0)	0.951 ± 0.12^b^	0.551 ± 0.01^a^	0.741 ± 0.03^b^	0.448 ± 0.01^a^	1.101 ± 0.11^b^	0.718 ± 0.02^b^	0.801 ± 0.03^b^	1.361 ± 0.11^b^
Palmitic (C16:0)	32.511 ± 5.11^b^	20.501 ± 1.91^a^	27.421 ± 2.21^a^	23.511 ± 2.01^a^	48.791 ± 3.21^c^	36.361 ± 2.91^b^	43.021 ± 4.01^c^	33.611 ± 2.02^b^
Heptadecanoic (C17:0)	0.121 ± 0.03^b^	0.083 ± 0.01^b^	0.101 ± 0.06^b^	0.048 ± 0.01^a^	0.143 ± 0.05^c^	0.103 ± 0.01^b^	0.102 ± 0.01^b^	0.136 ± 0.03^b^
Stearic (C18:0)	2.191 ± 0.03^a^	2.901 ± 0.1^b^	1.691 ± 0.21^a^	2.671 ± 0.51^a^	1.881 ± 0.21^a^	3.411 ± 0.41^b^	1.911 ± 0.32^a^	4.021 ± 0.31^c^
Arachidic (C20:0)	1.921 ± 0.51^ab^	1.831 ± 0.11^ab^	1.381 ± 0.07^a^	0.941 ± 0.03^a^	2.261 ± 0.21^b^	2.441 ± 0.31^b^	1.761 ± 0.21^ab^	1.181 ± 0.21^a^
Docosanoic (C22:0)	0.721 ± 0.11^a^	0.819 ± 0.11^ab^	0.632 ± 0.09^a^	0.611 ± 0.21^a^	0.825 ± 0.11^ab^	0.931 ± 0.07^b^	0.989 ± 0.11^b^	1.023 ± 0.21^b^
Tricosanoic (C23:0)	0.131 ± 0.01^b^	0.053 ± 0.01^a^	0.128 ± 0.01^b^	0.044 ± 0.01^a^	0.164 ± 0.02^c^	0.077 ± 0.02^a^	0.149 ± 0.02*^bc^*	0.099 ± 0.04^b^
Pentacosanoic (C25:0)	0.073 ± 0.02^b^	0.035 ± 0.02^a^	0.086 ± 0.01^b^	0.041 ± 0.02^a^	0.101 ± 0.01^a^	0.071 ± 0.02^ab^	0.099 ± 0.02^b^	0.061 ± 0.02^ab^
Palmitoleic (C16:1)	0.105 ± 0.03^a^	0.128 ± 0.02^a^	0.091 ± 0.03^a^	0.082 ± 0.01^a^	0.162 ± 0.04^ab^	0.158 ± 0.01^ab^	0.154 ± 0.05^a^	0.236 ± 0.05^b^
Heptadecenoic (C17:1)	0.176 ± 0.05^a^	0.232 ± 0.08^b^	0.184 ± 0.04^a^	0.191 ± 0.05^a^	0.193 ± 0.04^a^	0.267 ± 0.08^b^	0.216 ± 0.01^b^	0.193 ± 0.06^a^
Oleic (C18:1)	48.951 ± 0.83^a^	55.301 ± 1.21^ab^	45.602 ± 0.36^a^	42.231 ± 0.19^a^	77.161 ± 0.71^c^	66.331 ± 1.61^b^	70.521 ± 1.3^b^	68.341 ± 0.72^b^
Linoleic (C18:2)	0.096 ± 0.01^b^	0.054 ± 0.02^a^	0.082 ± 0.01^b^	0.039 ± 0.01^a^	0.102 ± 0.01^b^	0.106 ± 0.01^b^	0.146 ± 0.03^c^	0.114 ± 0.01^c^
Linolenic (C18:3 ω-3)	0.023 ± 0.01^a^	0.019 ± 0.01^b^	0.082 ± 0.05^b^	0.031 ± 0.01^a^	0.032 ± 0.01^a^	0.026 ± 0.01^a^	0.091 ± 0.02^b^	0.039 ± 0.02^a^
Eicosenoic (C20:1)	0.107 ± 0.02^ab^	0.067 ± 0.01^a^	0.081 ± 0.01^a^	0.069 ± 0.01^a^	0.141 ± 0.05^b^	0.102 ± 0.02^ab^	0.155 ± 0.01^a^	0.097 ± 0.01^a^
Tetracosenoic (C24:1)	0.018 ± 0.01^a^	0.011 ± 0.01^a^	0.015 ± 0.01^a^	0.009 ± 0.01^a^	0.025 ± 0.01^b^	0.025 ± 0.01^b^	0.029 ± 0.01^b^	0.022 ± 0.01^ab^

*Values are represented as the mean of 3 replicates (mean ± S.D.). The different letters indicate significant difference between groups in each row (p < 0.05).*

Additionally, the old and young leaves of C4 plants exhibited a negative response to the individual treatment with In_2_O_3_-NPs, which were significantly decreased in comparison to untreated control plants (Table 4). However, significant increments in arachidic, docosanoic, tricosanoic, and palmitoleic acids (increased by about 30–50%) were observed in only young leaves of C4 plants (Table 4). So, from the results it is appeared that the young leaves of C4 plants are more sensitive to the individual treatment of In_2_O_3_-NPs than the old leaves. Regarding the treatment with eCO_2_, it greatly induced the accumulation of palmitic, heptadecanoic, linoleic, tetracosanoic, arachidic, pentacosanoic, and eicosenoic (increased by about 20–50%), as well as docosanoic and oleic acids in both old and young leaves of C4 plants. Stearic and linoleic acids were enhanced only in the old leaves, while myristic acid was increased only in the young leaves of C4 plants. More interestingly, the combined treatment of C4 plants (in both old and young leaves) with In_2_O_3_-NPs and eCO_2_ remarkably enhanced the accumulation of heptadecanoic, arachidic, and palmitoleic acids, while pentacosanoic and linolenic acids were significantly reduced when compared with treated control plants grown under normal levels of CO_2_ (Table 4). By comparing both old and young leaves to each other, the levels of myristic, palmitic, docosanoic, oleic, eicosenoic, and tetracosenoic acids were significantly enhanced in only the young leaves of C4 plants. Therefore, in C4 plants, the combined effect of In_2_O_3_-NPs and eCO_2_ seemed to be more obvious on the young leaves than the old ones.

**TABLE 4:** Effect of the co-application of future climate CO_2_ and In_2_O_3_-NPs upon the fatty acids profile of both old and young leaves of C4 plants.

	C4 Plants				C4 Plants			
	Old leaves		Young leaves		Old leaves		Young leaves	
	aCO_2_	aCO_2_ + In_2_O_3_-NPs	aCO_2_	aCO_2_ + In_2_O_3_-NPs	eCO_2_	eCO_2_ + In_2_O_3_-NPs	eCO_2_	eCO_2_ + In_2_O_3_-NPs
Myristic (C14:0)	1.021 ± 0.09^b^	0.607 ± 0.02^ab^	0.731 ± 0.01^ab^	0.453 ± 0.01^a^	1.074 ± 0.04^b^	0.881 ± 0.02^ab^	0.915 ± 0.01^ab^	1.761 ± 0.08^b^
Palmitic (C16:0)	32.271 ± 5.01^b^	19.321 ± 2.11^a^	26.741 ± 0.61^a^	22.641 ± 2.11^a^	38.171 ± 0.91^b^	24.281 ± 2.41^a^	34.461 ± 3.11^b^	50.861 ± 1.11^c^
Heptadecanoic (C17:0)	0.073 ± 0.02^ab^	0.049 ± 0.01^a^	0.071 ± 0.05^ab^	0.057 ± 0.01^a^	0.114 ± 0.01^b^	0.094 ± 0.01^b^	0.095 ± 0.01^b^	0.161 ± 0.02^c^
Stearic (C18:0)	2.211 ± 0.41^a^	2.271 ± 0.31^a^	2.551 ± 0.41^ab^	1.811 ± 0.21^a^	2.815 ± 0.31^b^	3.421 ± 0.51^c^	2.481 ± 0.41^a^	5.581 ± 0.71^d^
Arachidic (C20:0)	1.281 ± 0.25^a^	1.461 ± 0.09^b^	1.071 ± 0.06^a^	1.511 ± 0.11^b^	1.331 ± 0.08^a^	1.531 ± 0.12^b^	1.271 ± 0.07^a^	1.761 ± 0.19^b^
Docosanoic (C22:0)	0.833 ± 0.11^b^	0.685 ± 0.11^a^	0.576 ± 0.11^a^	0.663 ± 0.21^a^	0.718 ± 0.12^b^	0.868 ± 0.11^b^	0.565 ± 0.08^a^	1.361 ± 0.21^c^
Tricosanoic (C23:0)	0.049 ± 0.01^a^	0.032 ± 0.01^a^	0.037 ± 0.01^a^	0.042 ± 0.01^a^	0.094 ± 0.01^a^	0.077 ± 0.01^b^	0.069 ± 0.01^b^	0.108 ± 0.04^b^
Pentacosanoic (C25:0)	0.074 ± 0.02^b^	0.034 ± 0.02^a^	0.077 ± 0.01^b^	0.051 ± 0.01^a^	0.087 ± 0.01^b^	0.045 ± 0.01^a^	0.094 ± 0.02^b^	0.064 ± 0.01^a^
Palmitoleic (C16:1)	0.129 ± 0.02^b^	0.103 ± 0.03^a^	0.087 ± 0.01^a^	0.105 ± 0.02^a^	0.254 ± 0.07^c^	0.225 ± 0.06^c^	0.144 ± 0.05^b^	0.265 ± 0.05^c^
Heptadecenoic (C17:1)	0.222 ± 0.07^a^	0.183 ± 0.04^a^	0.187 ± 0.05^a^	0.195 ± 0.05^a^	0.244 ± 0.04^b^	0.237 ± 0.02^b^	0.215 ± 0.06^b^	0.322 ± 0.09^c^
Oleic (C18:1)	60.191 ± 2.31^b^	48.911 ± 3.04^a^	53.881 ± 5.19^a^	42.491 ± 5.21^a^	61.471 ± 1.61^ab^	59.611 ± 3.31^b^	55.611 ± 2.81^a^	94.641 ± 6.07^c^
Linoleic (C18:2)	0.083 ± 0.01^b^	0.027 ± 0.01^a^	0.069 ± 0.02^ab^	0.043 ± 0.02^a^	0.102 ± 0.01^b^	0.102 ± 0.01^a^	0.056 ± 0.01^a^	0.056 ± 0.02^a^
Linolenic (C18:3 ω-3)	0.021 ± 0.01^a^	0.015 ± 0.01^a^	0.035 ± 0.01^b^	0.023 ± 0.01^a^	0.032 ± 0.01^b^	0.022 ± 0.01^a^	0.049 ± 0.01^c^	0.034 ± 0.01^a^
Eicosenoic (C20:1)	0.124 ± 0.05^b^	0.073 ± 0.01^a^	0.083 ± 0.01^a^	0.069 ± 0.01^a^	0.166 ± 0.01^b^	0.093 ± 0.01^a^	0.101 ± 0.01^a^	0.144 ± 0.01^b^
Tetracosenoic (C24:1)	0.024 ± 0.01^b^	0.009 ± 0.01^a^	0.023 ± 0.02^b^	0.019 ± 0.01^a^	0.038 ± 0.02^a^	0.013 ± 0.01^a^	0.034 ± 0.03^c^	0.038 ± 0.01^c^

*Values are represented as the mean of 3 replicates (mean ± S.D.). The different letters indicate significant difference between groups in each row (p < 0.05).*

#### Polyamines’ Metabolism

**FIGURE 5:**
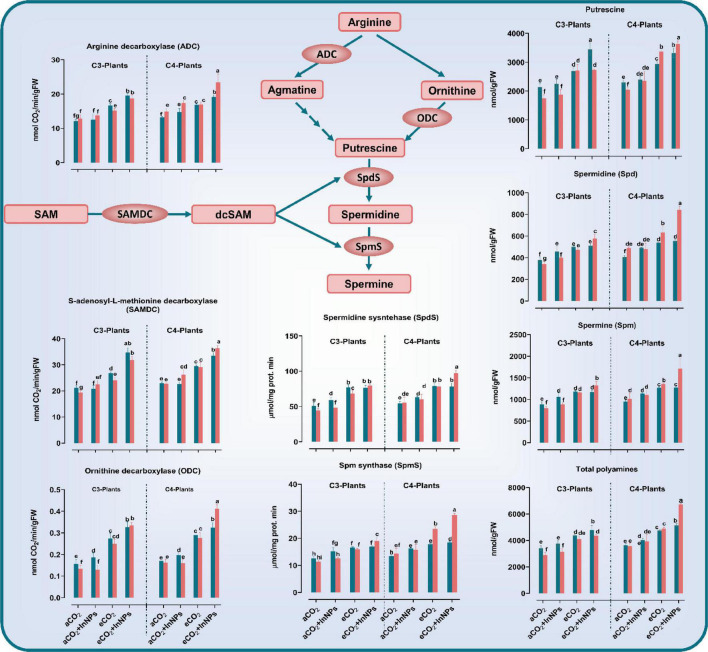
Effect of the co-application of future climate CO_2_ and In_2_O_3_-NPs upon polyamine metabolism in both old and young leaves of C3 and C4 plants. Four biological replicates are used to measure each value. The bars on the columns represent error bars (SE). One way ANOVA test (*P* < 0.05; *n* = 4) was applied to show the significant difference between groups. Different letters showed the significant difference between treatments.

In a decarboxylation reaction, ornithine or arginine is converted to putrescine, a common polyamine, in a reaction that is catalyzed by ornithine decarboxylase (ODC) or arginine decarboxylase (ADC) (Alcázar et al., 2010; Bitrián et al., 2012). Our results declared that the treatment with eCO_2_ either alone or in combination with In_2_O_3_-NPs caused a remarkable accumulation in the levels of putrescine (Put) as well as spermine (Spm) and spermidine (Spd) in addition to their metabolic enzymes (arginine decarboxylase; ADC, ornithine decarboxylase ODC, and *S*-adenosylmethionine decarboxylase; SAMDC) in the old and young leaves of both C3 and C4 plants (Figure 5). The individual treatment of C3 plants (at both old and young leaves) with In_2_O_3_-NPs led to significant elevations in Put, Spd, Spm, and their biosynthetic enzymes i.e., ADC and SAMDC [increased by about 10–20% as compared to the untreated control plants (Figure 5)]. Such an enhancing effect was more noticeable in the old leaves, whereas ODC was not affected in the young leaves. In addition, both old and young leaves were observed to be equally affected by the sole treatment with eCO_2_, leading to higher increments in Put, Spd, Spm, ADC, SAMDC (by about 30–50%), and ODC (∼100% increment) in comparison to control plants. The combined treatment of In_2_O_3_-NPs and eCO_2_ has also positively affected the levels of Put, Spm, Spd, arginine decarboxylase, and *S*-adenosylmethionine decarboxylase (increased by about 30–50%) in the old leaves of C3 plants when compared to control plants (Figure 5). On the other hand, the young leaves had a higher content of ODC that increased by about 300% relative to the control plants.

Regarding C4 plants, the sole treatment with In_2_O_3_-NPs caused a noticeable elevation in the levels of Put, Spd, Spm, ADC, and SAMDC in both old and young leaves. Meanwhile, ODC was not affected in young leaves, thus, the In_2_O_3_-NPs-induced effect was more obvious on young leaves than the old ones. Likewise, both old and young leaves equally responded to the individual treatment with eCO_2_, resulting in significant increases in Put, Spd, Spm, ADC, and SAMDC as well as the activity of ODC that increased by about 100% when compared with untreated control plants. The interactive effect imposed by In_2_O_3_-NPs and eCO_2_ was almost similar in both old and young leaves which accumulated higher levels of Put, Spd, Spm, ADC, SAMDC (increased by about 30–70%), and ODC that strikingly increased (∼200% increment) when compared to control plants.

## Discussion

Contamination of soil with NPs significantly decreases agricultural supply even though demands for improving crop productivity are increasing. Achieving this demand is sure to be a challenge with respect to future climatic changes and environmental pollutions with NPs. It is known that increased CO_2_ levels mitigate effects, however, it is not known whether this is the case for NPs. This study will shed light on the In_2_O_3_-NPs stress mitigating impact of eCO_2_. Thus, it will provide a scientific basis for optimizing eCO_2_ management for maintaining stable sorghum and maize production in In_2_O_3_-NP-contaminated soils.

### How Is Growth Mitigated by Elevated CO_2_ in Old and Young Leaves of Both C3 and C4 Plants?

In_2_O_3_-NPs significantly retarded the growth of both young and old leaves of C3 and C4 plants. The observed decreases in wheat and sorghum growth may be ascribed to the negative impact of In_2_O_3_-NPs on the vital biological activities such as cell division, nutrient uptake, photosynthesis, and respiration (Rastogi et al., 2017). This harmful effect could be due to the potency of In_2_O_3_-NPs to penetrate the cell and elicit molecular and cellular events in the cell (Buzea et al., 2007) including an increase in the cell wall rigidity, diminishing the cell wall growth, and finally cell rupturing (Kopittke et al., 2009). In line with our findings, the photosynthesis of wheat and maize showed a remarkable retardation after treatment with NiO and HgO nanoparticles (Saleh et al., 2019; AbdElgawad et al., 2020). Also, in nanoparticles strikingly less nutrient uptake in rice plants was observed due their adverse effect on the nutrient translocation to the shoots (Syu et al., 2017).

Besides being a prospective tool that enhances plant growth (Shabbaj et al., 2021a), eCO_2_ reduced the hazardous impact of In_2_O_3_-NPs. This alleviative impact of eCO_2_ was demonstrated in the recovery of plant growth and photosynthesis (Figure 1). Generally, C4 plants respond better to eCO_2_ at growth level. Consistently, photosynthetic efficiency was highly augmented in In_2_O_3_-contaminted old and young leaves In line with our findings, eCO_2_, within the physiological range, caused a remarkable restoration for the crops’ growth by enhancing their photosynthetic carbon assimilation and, accordingly, carbohydrate partitioning (Shabbaj et al., 2021a). To improve photosynthesis machinery, eCO_2_ is known to upregulate the RuBisco activity in different plant species under NPs stress conditions (AbdElgawad et al., 2020; Selim et al., 2021a). Increased CO_2_ substrate availability for RuBisco instantaneously inhibits photorespiration (Dusenge et al., 2019).

### How eCO_2_ Orchestrated N and C Metabolism in Stressed C3 and C4 Plants to Improve Indium Oxide Nanoparticles Stress Tolerance?

Studies investigating C3 and C4 species responses to climate conditions have so far mostly addressed changes in biomass, photosynthesis, and nutrient relations (Drake, 2014). Comparatively, much less is known about the effects of In_2_O_3_-NPs and/or eCO_2_ and associated heavy metal NPs stress in C3 and C4 at the carbon and nitrogen metabolism level. Like other heavy metal nanoparticles, In_2_O_3_-NPs threaten plant growth by retarding vital metabolic processes such as carbon and nitrogen partitioning (Rahoui et al., 2015). Our results revealed a remarkable alternation in carbon and nitrogen assimilation in both sorghum and wheat, particularly in young leaves. For instance, a reduction in starch level and an elevation of sucrose metabolizing enzymes was observed. It is well known that soluble sugars such as glucose and sucrose are the most pivotal structural and energetic organic compounds in higher plants (Shabbaj et al., 2021b). Besides being osmoregulators, sugars possess obvious ROS scavenging properties (Van den Ende, 2013).

Photosynthesis products and some of their derivatives play dual roles as metabolic intermediates and signaling molecules that influence plant cell metabolism (Ainsworth and Rogers, 2007). For instance, sugars are involved in the organic acids’ biosynthesis. Here, growing of C3 and C4 plants under In_2_O_3_-NPs accumulated organic acids, particularly in young leaves of C4, indicated their role in reducing uptake by plant roots and restricting their translocation to leaves (Panfili et al., 2009). Organic acids also act as stored pools of fixed carbon (Igamberdiev and Eprintsev, 2016), and as a bridge to connect C and N metabolism together such as N assimilation and amino acid biosynthesis (Raven and Farquhar, 1990). In addition to organic acid intermediates, amino acids biosynthesis also needs energy derived from sugar catabolism during dark respiration (Bolton, 2009). In turn, the accumulated amino acids, e.g., proline, improved plant tolerance to environmental challenges (Shabbaj et al., 2021b). Several amino acids can also act as substrate to produce polyamines, for instance the diamine Put is synthesized directly from ornithine by ODC or from arginine by ADC (Pal Bais and Ravishankar, 2002). Polyamines retrieve the different environmental abiotic stresses, including metal and metalloid stresses in plants (Hasanuzzaman et al., 2019). Besides being essential for plant growth and development, polyamines are crucial in maintaining the integrity of nucleic acids and ensuring their translocation (Cason et al., 2003). Sugars also provide substrates for *de novo* fatty acid biosynthesis (Zhai et al., 2021). Under environmental stress conditions, fatty acids mainly contribute to maintaining structural integrity and supplying energy for several plant metabolic activities. This is consistent with the elevation in the levels of tocopherols that scavenge ROS to preserve polyunsaturated fatty acids from lipid peroxidation (Krieger-Liszkay and Trebst, 2006). Contamination with In_2_O_3_-NPs caused a noticeable reduction in the levels of fatty acids, especially in young leaves of both C3 plants.

On the other hand, maintaining the photosynthetic machinery in stressed plants under eCO_2_ is very essential to maintain a continuous supply with non-structural carbohydrates that play a crucial role in enhancing plant tolerance with the continuous supply of amino, organic, and fatty acids (Thompson et al., 2017). eCO_2_ in combination with In_2_O_3_-NPs significantly reduced the sucrose levels with a concomitant increase in starch levels. In parallel with this finding, a noticeable reduction in the activities of sucrose-P-synthase in both C3 and C4 plants, particularly the young leaves of C4 plants, was observed. The accumulation of soluble sugars rather than storage sugars could proclaim the retrieval of the osmotic imbalance triggered by In_2_O_3_-NPs. In line with our results, the recovery of tomato leaves from salt stress was accompanied by low levels of sucrose and hexose with concomitant elevation in starch levels (Khelil et al., 2007).

To reduce In_2_O_3_-NPs uptake by plant, soluble sugars may be consumed under eCO_2_ to produce organic acids through respiration. Here, succinate and fumarate levels were induced, particularly in the old leaves of C3 plants, when treated with eCO_2_ under In_2_O_3_-NPs. Increased levels of succinate and fumarate can be explained by the ability of eCO_2_ to boost succinate dehydrogenase activity (Igamberdiev and Eprintsev, 2016). eCO_2_-induced organic acids’ accumulation contributes to manipulating the energy and redox status in the cell, therefore supplying the cell with the required precursors for the amino acids’ biosynthesis. Besides being essential for protein biosynthesis, amino acids are also involved in a wide array of osmolytes to maintain cellular turgor pressure (Suprasanna et al., 2016). Notably, eCO_2_ provoked the levels of glutamate and proline in both C3 and C4 crops, particularly young leaves of C3 plants, under In_2_O_3_-NPs contamination.

Another stress related amino acid, proline, is a crucial osmoregulator that enhances cell integrity and maintains cellular structure and enzymatic activity (Madany et al., 2020). A rate limiting enzyme denoted as P5CS can modulate proline biosynthesis from either ornithine or glutamate pathways. eCO_2_ clearly triggered both glutamine and ornithine pathways in old leaves and to a higher extent in young leaves of wheat In_2_O_3_-NPs stressed plants, however, ornithine pathway seems to be more abundant in both old and young leaves of C4 plants equally. This enhancement in the activities of proline biosynthetic enzymes was concomitant with a remarkable reduction in the activities of biodegrading enzymes (PRODH), particularly in young leaves of C4 plants.

One of the mechanisms involved in the stress mitigation impact of eCO_2_ is the biosynthesis of polyamines. Of note, the aforementioned polyamines have been proven to scavenge free radicles *in vitro* (Sengupta et al., 2015). Under stress conditions, polyamines can also act as signaling molecules that can modulate the ion homeostasis and consequently organize the ion transportation by interacting with the ion channels (Podlešáková et al., 2019). They also play a pivotal role in diminishing ROS accumulation and preventing lipid peroxidation, hence stabilizing the membrane (Liu et al., 2007; Spormann et al., 2021). In this context, Quartacci et al. (2001) found that the oxidative damage caused by Cd^2+^ or Cu^2+^ was ameliorated by the exogenous application of Spm upon wheat leaves by maintaining the membrane integrity via stabilizing lipids and avoiding solute leakage.

The co-application of future climate eCO_2_ with In_2_O_3_-NPs strikingly enhanced the accumulation of fatty acids, particularly unsaturated fatty acids (18:1, 18:2, and 18:3), in young leaves of both C3 and C4 crops. The increased levels of oleic acid (18:1) could contribute to restoring the plant defense signaling (Nandi et al., 2003). In addition, the elevation in polyunsaturated fatty acids and tocopherols levels in response to eCO_2_ is a good sign of membrane stability under In_2_O_3_-NPs pollution. On the other side, fatty acids provide several plant metabolic processes with energy and structural integrity, and so play a crucial role in regulating signal transduction under different environmental challenges.

### Young and Old Leaves of C3 and C4 Plants Respond Differently to Indium Oxide Nanoparticles Stress and eCO_2_

**FIGURE 6:**
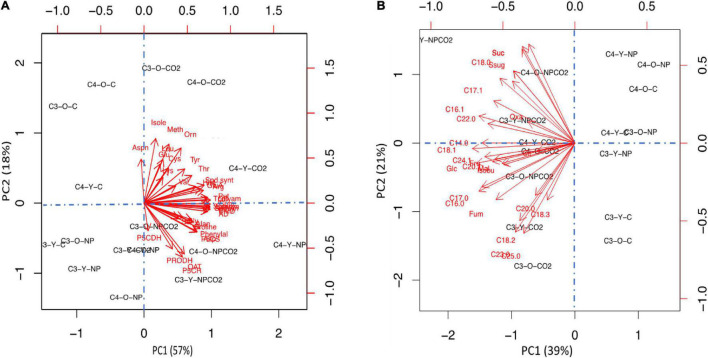
Principal component analysis (PCA) to investigate the variability of data for both carbon metabolites **(A)** and nitrogen metabolites **(B)**. The arrows showed which variables are most correlated with the principal components (PCs). The link between variables is defined by the arrow proximity.

To get a better view on the differential responses to the effect of In_2_O_3_-NPs stress and eCO_2_, we applied principal component analysis (PCA) using all measured nitrogen-related metabolites (Figure 6A) and carbon-related metabolites (Figure 6B). At nitrogen metabolites level, principal component 1 (PC1) and principal component 2 (PC2) explained 57 and 18% of the total variance, respectively. However, at carbon metabolites level, principal component 1 (PC1) and principal component 2 (PC2) explained 39 and 21% of the total variance, respectively. The PCA shows a clear separation in Cr treatment with PC1. In general, the PCA analysis revealed a clear separation of the two treatments of eCO_2_ (either alone or in combination with In_2_O_3_-NPs) on development in the two plant species in either old or young leaves (Figure 6A). At nitrogen metabolism level, eCO_2_ clearly induced more levels of amino acids and polyamines under control or stress conditions, which explain 57% of the variation (Figure 6A, PC1). Moreover, the old leaves of both C3 and C4 plants showed more specific responses, particular under the control conditions. Similar, the second PCA (Figure 6B) showed that the effect of ambient CO_2_ on carbon metabolism was lower compared to elevated CO_2_ levels. The main drivers for PC1 (39%) were unsaturated fatty acid and soluble sugars.

For species specific responses, we found the C4 data were clustered together and separated from C3 data across PC2 (21%, Figure 6B). The pattern was more diffuse for leaf types. For instance, our PCA analysis declared that the increment in sucrose was species dependent as its level was increased in both old and young leaves of C4 plants in response to eCO_2_ under In_2_O_3_-NPs treatment. At the growth level, fewer reductions were observed for C4, indicating that C4 crops are more tolerant to heavy metal stress than C3 crops (Srivastava et al., 2012). This enhanced tolerance of C4 plants could be attributed to the efficient photosynthetic activity and so enhance growth rates as compared to C3 crops (AbdElgawad et al., 2020). Together this suggests that the primary processes affected by elevated CO_2_ under abiotic stress conditions may not be identical in young and old leaves of both C3 and C4 species groups.

## Conclusion

Our objective in this study was to test the hypothesis that eCO_2_ can alleviate the deleterious impact of In_2_O_3_-NPs upon carbon and nitrogen metabolism of both C3 and C4 plants. Our findings show that eCO_2_ can regulate both carbon and nitrogen metabolism in such a way that improves the plant tolerance to pollution with In_2_O_3_-NPs. This improvement was clearly embodied in enhancing the photosynthetic efficiency that was accompanied with a concomitant improvement in the plant biomass under both clean and polluted conditions (Supplementary Figure 1). This mitigative effect not only restricted photosynthesis and biomass, but also enhanced the biosynthesis and accumulation of sugars, organic acids, amino acids, fatty acids, and polyamines. Overall, the present study, for the first time, offers an insight upon the ability of eCO_2_ to manipulate carbon and nitrogen metabolism in both C3 and C4 plants to tolerate contamination with heavy metal, especially In_2_O_3_-NPs.

## Data Availability Statement

The original contributions presented in the study are included in the article/Supplementary Material, further inquiries can be directed to the corresponding author.

## Author Contributions

MMYM and HA: conceptualization, methodology, resources, data curation, and writing—original draft preparation. MMYM, HA, and IIS: software and data validation. IIS and MAB: formal analysis. IIS, HA, MMYM, MAB, and AT: investigation. MMYM, HA, IIS, and AT: writing—review and editing. IIS: visualization. IIS, AT, and MAB: project administration. IIS and AT: funding acquisition. All authors have read and agreed to the published version of the manuscript.

## Conflict of Interest

The authors declare that the research was conducted in the absence of any commercial or financial relationships that could be construed as a potential conflict of interest.

## Publisher’s Note

All claims expressed in this article are solely those of the authors and do not necessarily represent those of their affiliated organizations, or those of the publisher, the editors and the reviewers. Any product that may be evaluated in this article, or claim that may be made by its manufacturer, is not guaranteed or endorsed by the publisher.

## Footnotes

1
https://www.americanelements.com/indium-oxide-nanoparticles-nanopowder-1312-43-2
