# Supplementary material for: CO2 Enrichment Differentially Upregulated Sugar, Proline, and Polyamine Metabolism in Young and Old Leaves of Wheat and Sorghum to Mitigate Indium Oxide Nanoparticles Toxicity
Source: Front Plant Sci. 2022 May 3;13:843771. doi: 10.3389/fpls.2022.843771 (PMC9112856; doi:10.3389/fpls.2022.843771)
Supplement: Supplementary file 1 [file Data_Sheet_1.PDF]

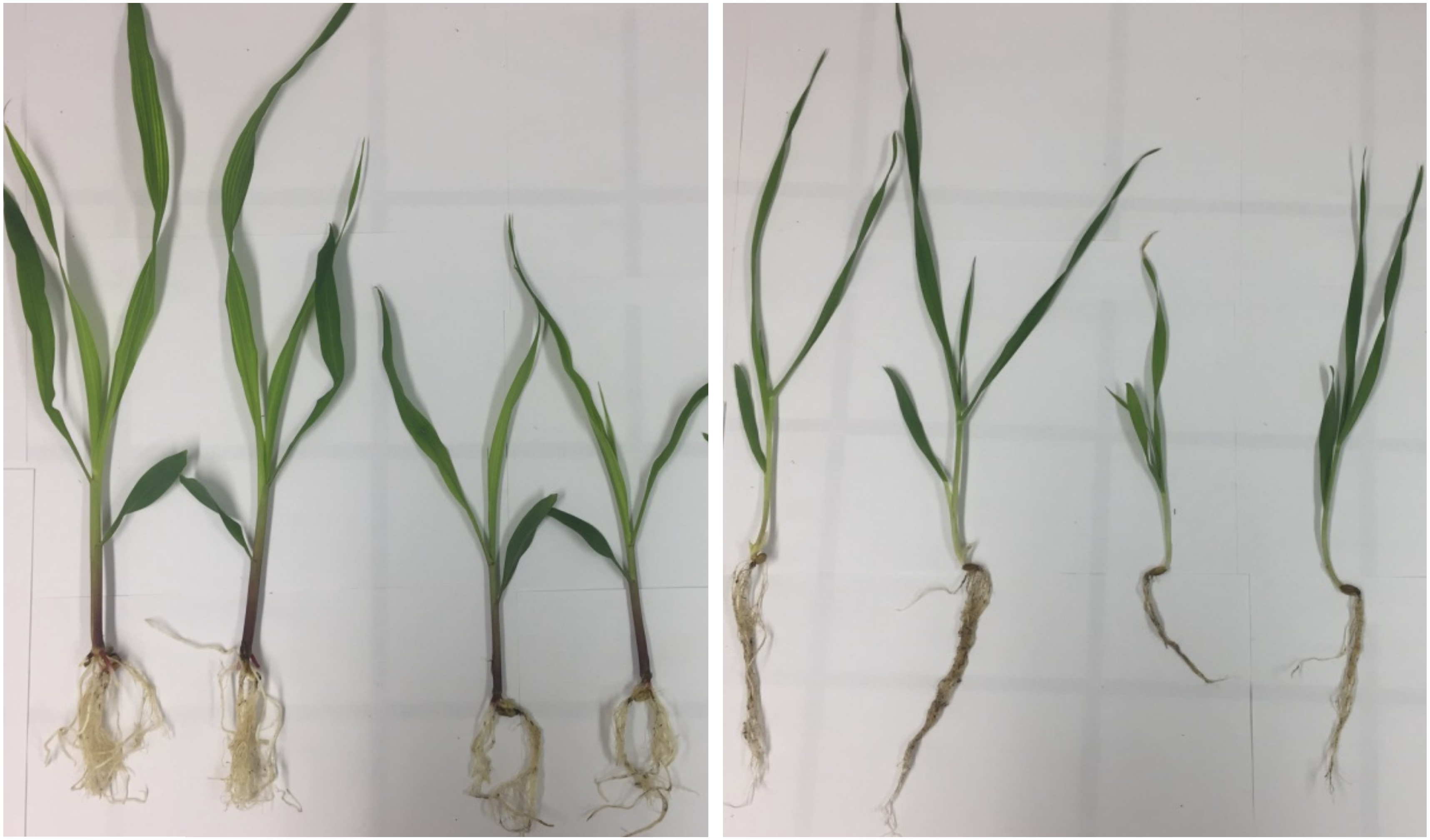

Sorghum Control | Sorghum eCO_2 | Sorghum In_2O_3NPs | Sorghum $In_2O_3NPs+eCO_2$ | Wheat Control | Wheat eCO_2 | Wheat In_2O_3NPs | Wheat $In_2O_3NPs+eCO_2$

Figure S1:
The effect of elevated CO_2 either alone or in combination with In_2O_3-NPs upon the growth of both sorghum and wheat plants.
